# Supplementary material for: Race and ethnicity do not impact eligibility for remdesivir: A single-center experience
Source: PLoS One. 2021 May 6;16(5):e0250735. doi: 10.1371/journal.pone.0250735 (PMC8101938; doi:10.1371/journal.pone.0250735)
Supplement: S1 File — (DOCX) [file pone.0250735.s001.docx]

S1 File.

Immunosuppressed criteria:

- Cancer treatment within the past one year
- Current use of immune suppressive drugs: TNF-alpha inhibitors, prednisone greater than 20 mg for 2 weeks, tacrolimus, mycophenolate, sirolimus, rituximab, vedolizumab, abatacept, eculizumab, vedolizumab
- Bone marrow or solid organ transplant
- HIV positive regardless of CD4 count
